# The Active Room: Freud’s Office and the Egyptian Tomb

**DOI:** 10.3389/fpsyg.2020.01547

**Published:** 2020-07-28

**Authors:** Julia K. Schroeder

**Affiliations:** Department of German, University of California, Berkeley, Berkeley, CA, United States

**Keywords:** Sigmund Freud, office design, psychoanalysis, work environment, active room, therapy, extended mind, ancient Egypt

## Abstract

The present study examines the striking similarities between the architectural design and spatial composition of the ancient Egyptian tomb and Sigmund Freud’s office at Berggasse 19 in Vienna, Austria. I argue that the Egyptian tomb elements represented within Freud’s office permitted the enclosed space to play an active role in his psychoanalysis sessions. I supplement this argument by analyzing the office’s spatial and architectural arrangements in relation to ancient Egyptian architectural frameworks, psychoanalytic container theory (Freud, Danze, and Quinodoz), and Freud’s archeological metaphor model. This study contributes to the greater body of work on architecture as an active entity, psychoanalysis, and ancient Egyptian history.

*I then made some short observations upon the psychological differences between the conscious and unconscious, and upon the fact that everything conscious was subject to a process of wearing-away, while what was unconscious was relatively unchangeable; and I illustrated my remarks by pointing to the antiques standing in my room. They were, in fact, I said, only objects found in a tomb, and their burial had been their preservation.* – Sigmund Freud, *Notes Upon a Case of Obsessional Neurosis*

## Freud’s Office and the Egyptian Tomb

In his 1937 essay “Constructions in Analysis,” Sigmund Freud related King Tutankhamun’s well-preserved Egyptian tomb to his psychoanalytic practice. Upon closer inspection, it appears that Freud’s office floor plan nearly matches Egyptian 18th Dynasty tomb floor plans, including that of King Tutankhamun. In the tomb and office, the space that held the body was composed of conjoining, externally sealed off rooms. Representations of essential Egyptian tomb elements and furniture were also included inside Freud’s enclosed, tomb-like office.

The office’s spatial and physical design appears to have permitted the patient to participate in free association and, in turn, overcome past traumas. The Egyptian tomb’s spatial and physical design was considered to permit the mummy’s spirit to transcend the body and transition into the afterlife. Following contemporary, psychoanalytic “active room” theory (Danze, 2005) and “active container” theory (Quinodoz, 1992), Freud’s office and the ancient Egyptian tomb were active rooms–closed-off spaces that significantly contributed to the contained individual’s psychic transformation.

The office’s Egyptian elements and tomb-like architecture appear to illuminate the profound influence Egyptian culture and civilization had on Freud’s psychoanalytic setting and practice. Freud’s iconic, tomb-like office laid the foundation for the contemporary emphasis on psychoanalytic office design and architecture. The analyst’s room retains its active role in psychoanalysis sessions. The present analysis contributes to the greater body of work on architecture as an active entity, psychoanalysis, and ancient Egyptian history. Furthermore, this study builds upon the article “Berggasse 19: Inside Freud’s Office” in which Diana Fuss and Joel Sanders posit Freud’s “labor of self-entombment” within his office space (Fuss and Sanders, 2015, p. 3). The present article extends beyond Freud’s self-entombment and posits that the tomb-like elements and architecture in Freud’s office, moreover, played a central role in facilitating the transformation and liberation of the patient’s psyche.

## Archeology and Architecture

**FIGURE 1:**
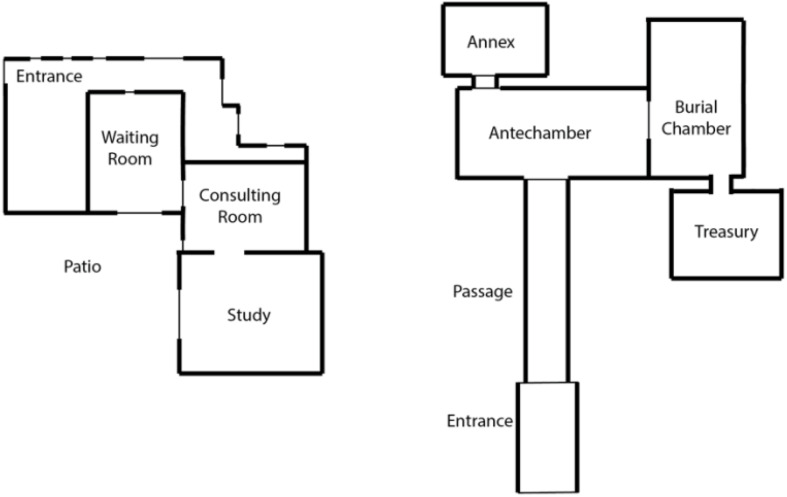
The **left** image shows a floor plan of Sigmund Freud’s office at Berggasse 19 in Vienna, Austria. Note that the consulting room and study are conjoined by an open doorway. The door between the waiting room and the consulting room is sealed. The **right** image shows a floor plan of King Tutankhamun’s tomb. Note that the burial chamber and treasury are conjoined by an open doorway. The door between the antechamber and burial chamber is sealed. (Figure 1 by Julia K. Schroeder).

**FIGURE 2:**
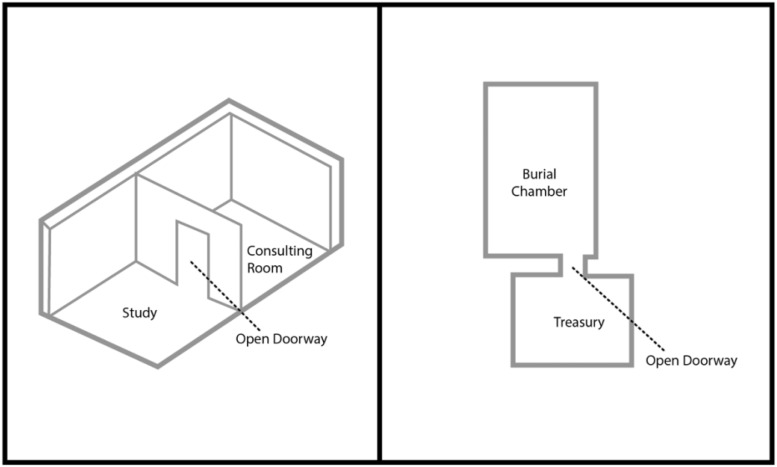
The **left** image shows a three-dimensional model of Freud’s office at Berggasse 19. The **right** image shows a model of King Tutankhamun’s tomb in Egypt’s Valley of the Kings. Note that each space is a single room. (Figure 2 by Julia K. Schroeder).

Freud’s office floor plan bears a strong resemblance to 18th Dynasty Egyptian tomb floor plans, including that of King Tutankhamun (see Figure 1). Freud’s office consisted of his consulting room and study, which formed a single room. In King Tutankhamun’s tomb, the burial chamber and treasury formed a single room. In Freud’s office and the Egyptian tomb, an open doorway connected the two spaces. Each room was a contained space complete with an open portal at its center (see Figure 2).

A comparison of the tomb and office floor plans may be viewed as Freud’s consulting room being in the position of the tomb’s burial chamber. In the burial chamber, the mummy lay in the sarcophagus. In Freud’s consulting room, the patient lay on the couch. Freud’s study was in the position of the tomb treasury. The mummy’s personal belongings and furniture were kept in the treasury. The majority of Freud’s Egyptian treasures and furniture were kept in his study. Commenting on Freud’s Egyptian treasures, Fuss and Sanders write that Freud assembled “antiquities that would transform his office into a veritable tomb” (Fuss and Sanders, 2015, p. 3). Augmenting this claim, in his memoirs, Freud’s patient Sergei Pankejeff remarked that the office “in no way reminded one of a doctor’s office … Here were all kinds of statuettes and other unusual objects, which even a layman recognized as archeological findings from ancient Egypt … Everything here … contributed to one’s feeling … of being sheltered” (Gardiner, 1973, p. 139).

The floor plans of Freud’s office and the Egyptian tomb reveal that each space’s external doors remained sealed. Freud’s office and the Egyptian tomb appear to be closed-off containers that significantly contributed to psychic and spiritual transformations, respectively. Each space was defined by its external, closed-off form and internal transformative function. These characteristics resonate with the contemporary psychoanalytic active container framework.

## Active Container

The Egyptian tomb and Freud’s office represent active containers. According to Swiss psychoanalyst Danielle Quinodoz’s (1992) article “The Psychoanalytic Setting as the Instrument of the Container Function,” the psychoanalyst’s office is an “active container.” In other words, the room “is not an inert vessel … but an active container which interacts dynamically with the [psychoanalysis] process” (Quinodoz, 1992, p. 629). The active container’s external features enable the patient to “gain access to the new relational world in which the unconscious psychical mechanisms begin to come alive and in which internal psychical reality becomes as real as external reality” (Quinodoz, 1992, p. 629). Freud’s office and the ancient Egyptian tomb were enclosed spaces that opened up new relational worlds for the mummy and patient. Each room held the body and permitted the mind to undergo a liberating, psychic transformation.

In the tomb, the mummy’s body rested in the sarcophagus as its spirit journeyed into the afterlife. In the office, the patient rested on Freud’s couch and journeyed into the realm of the unconscious. In the present study, the Egyptian afterlife serves as a metaphor for the unconscious processes accessed by the patient during free association. Each room liberated the mind, while the housed body remained physically in a resting position. The tomb-like architecture and spatial composition of Freud’s office stimulated the resting patient’s psyche and physical body. Freud himself drew connections between ancient tombs and psychoanalysis in his writings on the archeological metaphor.

## Freud’s Archeological Metaphor

Freud’s comparisons between psychoanalysis and archeology formed the basis of his archeological metaphor. This metaphor further reveals how his contained, tomb-like office played an active role in liberating the patient’s mind. In his writings, Freud linked his psychic excavations to tomb archeologist Heinrich Schliemann’s physical excavations. Drawing upon Schliemann’s excavation of Mycenae, Freud wrote in his essay “Female Sexuality”: “our insight into this early, pre-Oedipus, phase in girls comes to us as a surprise, like the discovery, in another field, of the Minoan–Mycenaean civilizations behind the civilization of Greece” (Freud, 1931, p. 226). Freud claimed the archeologist and the psychoanalyst were united by their shared mission to discover and preserve the past. The tomb and office actively aided the archeologist and the psychoanalyst in this shared endeavor. Freud’s passion for archeology resurfaces in his writings, office architecture, and book collection.

## Archeologist Heinrich Schliemann’s Influence on Freud

Freud cherished his copy of Heinrich Schliemann’s Mycenae and Tiryns archeology reports, which “were inscribed by him with the date of their purchase in the 1890s” (Freud Museum London, 2018, p. 1). In Schliemann’s (1878) publication entitled *Mycenae: A Narrative of Researches and Discoveries at Mycenae and Tiryns*, he described his excavation of these two ancient Greek cities. Throughout his descriptions of his 1878 Greek tomb excavations in Mycenae, Schliemann draws comparisons between Greek and Egyptian tomb architecture and elements. As stated in the preface written by the prime minister of England, William Ewart Gladstone, Schliemann’s work “affords a new indication of prehistoric relations between Mycenae and Egypt” (Schliemann, 1878, p. 7). In the 10th chapter on “the five Royal Tombs at Mycenae,” Schliemann notes the “whole household furniture, copper kettles, drinking vessels, and so forth of a rich warrior” were found in the tombs (Schliemann, 1878, p. 349). Commenting on these findings, Schliemann writes: “I hardly think it necessary to remind the reader of the custom of ancient Egypt of burying the dead with treasures, for all the collections of Egyptian antiquities in the world are produced from Egyptian tombs” (Schliemann, 1878, p. 349). In the Egyptian tomb and Freud’s office, the antiquities appeared to play an essential part in activating the rooms.

## Freud’s Tomb-Like Office

In 1909, Freud wrote that the antiquities found in his study and conjoint consulting room were “only objects found in a tomb” (Freud, 1909, p. 176). Freud might well have actively created a tomb-like atmosphere within his office. By 1938, Freud had collected more than 2,000 ancient objects. Approximately half of these objects were from ancient Egypt. In the 2005 article “An Architect’s View of Introspective Space: The Analytic Vessel,” architect and professor Elizabeth A. Danze delineates how such objects contribute to the experience within the psychoanalyst’s active container, which she terms an “active room.” Danze claims that the active room’s “objects, items, and art … might provide opportunities for association … These items are not neutral, but rather hold and present opportunities … they are ripe for symbolic interpretation” (Danze, 2005, p. 123). Freud’s comparisons between psychoanalysis and tomb archeology reveal the symbolic value of his antiquities and their active role in his practice.

In “Constructions in Analysis,” Freud related the resurfacing of intact memories in psychoanalysis to the excavation of King Tutankhamun’s intact tomb. Freud claimed analysts “are regularly met by a situation which with the archaeological object occurs only in such rare circumstances as those of Pompeii or of the tomb of Tutankhamun. All of the essentials are preserved; even things that seem completely forgotten are present” (Freud, 1937, p. 260). The tomb-like office not only resembled King Tutankhamun’s intact tomb but also provided optimal conditions for the analyst and patient to access and recover the buried past. The psyche’s buried past may be drawn to the surface within the active room.

## Space as Extension of the Psychic Apparatus

In each active room, space appears to represent an extension of the psyche. In 1938, Freud wrote that “space may be the projection of the extension of the psychical apparatus. No other derivation is possible … Psyche is extended” (Freud, 1938, p. 23). Freud seemed to conceptualize space as an active entity. The active room’s space may be conceived as forming a discursive force field around the analyst and the analysand. The force field was charged by the psychic energy of past and present, conscious and unconscious thought processes. Here, it is important to note that in Freud’s writings, the unconscious is often “ignorant of time, conserving its objects like an Egyptian tomb” (Deleuze and Guattari, 2004, p. 106). Much like the sealed off Egyptian tomb, the office’s “psychic force field” appears to have been reinforced by physical walls and closed doors.

In his *Introductory Lectures on Psychoanalysis*, Freud writes: “I have had the ordinary door between my waiting room and my office doubled and strengthened by a covering of felt … it is in his [the patient’s] interest not to be listened to while he is talking to the physician” (Freud, 1917, pp. 196–197). In Freud’s office, closing the soundproof doors activated the room by providing the patient with the security he needed to draw his unconscious memories to the surface. This security was enhanced by Freud in 1907, when he moved his study and consulting room to the back of the Berggasse 19 apartment. This further isolated the office from the Freud family’s living quarters. Contained by the office’s walls and closed, soundproof doors, the patient lying on Freud’s couch underwent psychic transformations as he accessed his unconscious past. Freud believed unconscious thoughts could be made conscious through spoken language. During free association, the patient’s spoken words permitted his unconscious thoughts to be unearthed and transformed into clear mental images. The words exiting the patient’s mouth may be compared with the words and spirit leaving the mummy’s mouth. Inside each sealed, active container, the resting body’s internal psychic reality was externalized through spoken language.

In ancient Egypt, the opening of the mummy’s mouth was a prerequisite for the deceased’s “passage to the beyond-for he must use his mouth to pronounce the names of the door-guardians and answer their questions, and he must speak at the judgment to prove himself worthy to enter the afterlife” (Taylor, 2010, p. 88). After the body was laid into the sarcophagus, a stone was traditionally placed on the mummy’s mouth. The stone enabled the mummy to speak during the judgment procedure and in the Land of the Dead. As Freud pointed out in his writings on the archeological metaphor: “Saxa loquuntur [‘Stones talk!’]” (Freud, 1896, p. 192).

Sealing the tomb permitted the mummy’s spirit to transcend the body and cross over into the Land of the Dead. In Freud’s office, the patient’s accessing of his unconscious permitted his metaphorical crossing over into the afterlife. The enclosed active rooms made it possible for the resting body to undergo psychic transformations. Each room’s central, open portal signified the space’s transformative potential. The patient’s and mummy’s psychic transformations connoted a rebirth within the room.

## Freud’s Tomb-Like Office Elements

The ancient Egyptian tomb architecture and the precise composition of the internal tomb elements initiated the mummy’s rebirth and transition into the afterlife. The inner realms of ancient Egyptian tombs included terracotta statuettes much like those on Freud’s desk. The Egyptian tomb murals painted on the durable, stone walls resembled Freud’s colorful, Egyptian paintings and stone plaques. The tomb mirrors strategically positioned near the mummy’s head mirrored the placement of Freud’s Etruscan mirrors. Freud’s couch found its double in the Egyptian sarcophagus. The tomb’s false door was comparable with Freud’s discrete, back exit door. Freud’s office and the Egyptian tomb were filled with vast amounts of personal treasures. Essential tomb artifacts, such as canopic jars, boxes, and mummy cloth, were also present within Freud’s tomb-like office. Freud’s oriental rugs may be seen as analogous to traditional linen mummy cloths. The office’s rugs and the tomb’s mummy cloths securely held and preserved the resting mummy’s and patient’s bodies. By taking a closer look at some of these elements, we find how each contributes to the room’s status as an active container.

## Rugs as Mummy Cloth

In Freud’s office, soft, Persian rugs were draped over and above the couch. The ornately patterned cloths enveloped and held Freud’s patients during psychoanalysis sessions. In a description of Freud’s office space, Donald W. Winnicott emphasizes the comfort generated by the patient’s designated space in the haptic consulting room. Winnicott observes: “the patient would be lying on a couch, that is to say comfortable … and probably a rug and some water would be available” (Winnicott, 1955, p. 285). In architecture, the haptic realm is “defined by sense of touch. When the materiality of the details forming an architectural space become evident, the haptic realm is opened up. Sensory experience is intensified; psychological dimensions are engaged” (Holl, 1996, p. 16). In Freud’s office, the tactile elements played an active role in opening the patient’s unconscious mind. The rugs stimulated the patient’s senses and psyche. The mummy laid bare his soul once protected by the haptic, linen cloths. Freud’s interest in Egyptian burial practices was reflected by the linen mummy cloths displayed in his office.

Freud showcased mummy cloth “made of flaxen cloth or pure linen” in his tomb-like office (Caminos, 1992, p. 338). The displayed cloth’s “21 warp threads and 11 woof threads to the centimeter” are in “agreement with the usual texture of Egyptian mummy linens” (Caminos, 1992, p. 338). These Egyptian mummy bandages were purchased by Freud around 1896 when he began collecting artworks and antiquities.

Although the year 1896 demarcates the death of Freud’s father, another pivotal date looms in the background. Freud was preparing for his own death when he began collecting Egyptian antiquities. On June 22, 1894, Freud wrote to his friend and colleague Wilhelm Fliess, “I shall go on suffering from various complaints for another 4 to 8 years with good and bad periods and then between forty and fifty perish very abruptly from a rupture of the heart” (Hunter, 1989, p. 99). Freud did not die during the predicted time frame. However, Freud’s father passed away.

Diana Fuss and Joel Sanders point out that in 1886 Freud’s father “fell fatally ill and died of heart failure … Freud apparently felt that his father died in his place” (Fuss and Sanders, 2015, p. 4). Fuss and Sanders claim that Freud’s preoccupation with his own death and his father’s death played a central role in his “death deliria” (Fuss and Sanders, 2015, p. 3). Fuss and Sanders write that “the lingering death of Jakob Freud is generally recognized as the emotional crisis that galvanized Freud’s compensatory interest in collecting” antiquities (Fuss and Sanders, 2015, p. 3).

Adding complexity to this generally accepted view, it seems relevant to point out that Freud’s interest in collecting ancient Egyptian funerary antiquities preceded his father’s death. In the years that Freud began dedicating himself to collecting tomb antiquities, he frequented the Louvre’s ancient Egyptian collection (1985), coined the term psychoanalysis (1986), wrote “The Aetiology of Hysteria” (1986), and began writing *On the Interpretation of Dreams* (1986). Each of these endeavors is haunted by Freud’s interest in ancient Egypt.

## Freud’s Early Egyptian Encounters

In 1885, while he was a student in Paris under Dr. Jean-Martin Charcot, Freud developed his first thoughts on what would evolve into psychoanalysis. Charcot’s lectures on hypnosis and hysteria in confluence with Freud’s visits to the Louvre were formative in his early conceptualizations of psychoanalysis. A year later, in 1886, Freud coined the term psychoanalysis “within the framework of a new theoretical understanding of the … etiology of hysteria” (Roazen, 1997, p. 39). Freud’s essay “The Aetiology of Hysteria” seems to reference ancient Egyptian tomb architecture in the section that describes an archeologist’s discovery of an ancient “treasure-house” bearing “numerous inscriptions,” which “yield undreamed-of-information” once “deciphered and translated” (Freud, 1896, p. 192). It is known that this particular text was “influenced by the school of Charcot” (Roazen, 1997, p. 39). Much of Charcot’s practice may be traced back to ancient Egypt.

Here, it is important to note that hypnosis (as performed by Charcot) was first practiced in ancient Egyptian sleep temples under the influence of Imhotep. Hysteria may also be traced back to ancient Egypt. In his office, Freud paid homage to the fathers of hypnosis. He showcased multiple Egyptian Imhotep statuettes and a reproduction of Pierre Albert-Brouillet’s engraving “La Leçon Clinique du Dr. Charcot.” Freud was deeply inspired by his encounters with Charcot and the ancient Egyptian ruins displayed in Paris.

In Paris, Freud frequented the Louvre’s Egyptian collection. On October 19, 1885, following his first visit to the Louvre, Freud wrote to his fiancée Martha Bernays: “I just had time for a fleeting glance at the Assyrian and Egyptian rooms, which I must visit again several times” (Freud, 1975, p. 173). In the letter, Freud also describes the Egyptian obelisk from Luxor displayed at Place de la Concorde. The letter reads:

Imagine a genuine obelisk, scribbled all over with the most beautiful birds’ heads, little seated men and other hieroglyphs, at least three thousand years older than the vulgar crowd round it, built in honor of the King whose name today only a few people can read and who, but for the monument, might be forgotten … For me these things have more historical than aesthetic interest (Freud, 1975, p. 173).

Freud’s Paris letters reveal early traces of his passion for ancient Egyptian antiquities as do his cherished copies of Schliemann’s work. Freud’s passion for collecting Egyptian funerary artifacts was sparked by the above events and may have been further amplified subsequently by his father’s death. Freud’s interest in ancient Egypt before his father’s death and heightened interest in the time of sorrow and dread after his father’s death seem to have both contributed to his creation of a tomb-like office space.

It is also useful to point out that in his letter, Freud emphasizes the “beautiful birds’ heads” (Freud, 1975, p. 173). In Egyptian tomb hieroglyphs, the direction the sparrow hawk faced indicated in which direction the text should be read. Freud’s later writings disclosed his awareness of this system. In *On the Interpretation of Dreams*, Freud wrote: “of sparrow hawks from an Egyptian tomb-relief” (Freud, 1900, p. 183). He noted: “to be able to read [Egyptian hieroglyphs] we … must depend upon the faces of the … birds” (Freud, 1900, p. 184).

Freud was particularly fascinated by the Egyptian writing system’s “vertical rows … considerations of beauty and proportion” (Freud, 1900, p. 184). The composition and organization of Freud’s office furniture and artifacts appear to also adhere to an organized system.

## Architecture, Space, and Composition

Freud was known to arrange his antiquities thematically in his case-like shelves and on his tables. In *The Psychopathology of Everyday Life*, Freud remarks, “shortage of space in my study has often forced me to handle a number of pottery and stone antiquities (of which I have a small collection) in the most uncomfortable positions, so that onlookers have expressed anxiety that I should knock something down and break it. That however, has never happened” (Freud, 1901, p. 55). Although crowded, the object arrangements in Freud’s tomb-like office followed a specific order that he knew well. Danze notes that “the position of the furniture … and the space between objects in the [active] room, establishes the sense of intimacy, familiarity, and safety” (Danze, 2005, p. 114).

In ancient Egypt, the spatial composition and orientation of tomb architecture, art, and furniture followed a specific scheme based on the greater environment. German Egyptologist Alexandra Verbovsek describes the basis of Egyptian architectural orientation and composition in the following excerpt:

[Egyptian] architecture or objects featured with images could–in a broader context–be integrated into comprehensive landscape conceptions which provide specific ways of viewing or axes of perception … For example, the alleys of sphinxes were visibly associated with particular buildings, assigned cult axes, and aligned the way toward temple entrances … guided actions and gave orientation within rooms (Verbovsek, 2014, p. 144).

In Freud’s office, furniture and object composition served as a means of orientation. The architectural scheme appeared to create telling axis lines within the space. The axis lines revealed what may be considered the room’s doubles.

**FIGURE 3:**
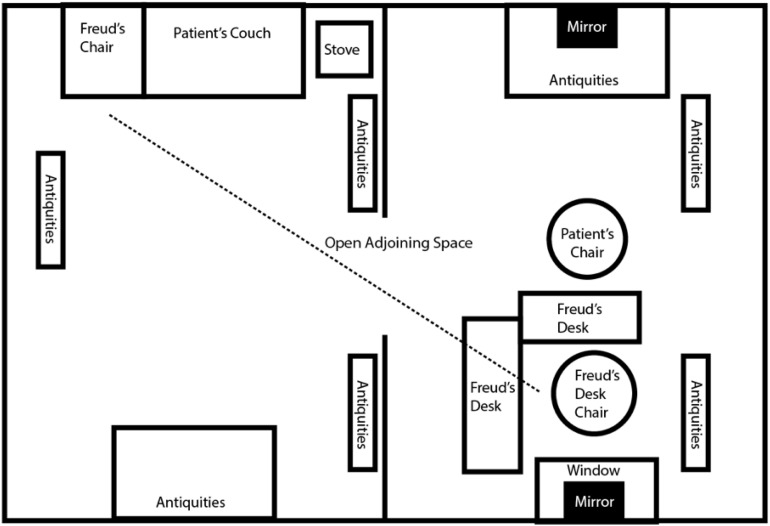
Depiction of Freud’s Office Architecture: Study **(left)** and Consulting Room **(right)**. If Freud directed his gaze diagonally across the room into his study from his consulting room chair, his study chair was directly in his line of vision (Figure 3 by Julia K. Schroeder).

If we draw a diagonal line through the central, open doorway across Freud’s room, we may connect the couch to his desk. We may also connect Freud’s consulting room chair to his anthropomorphic study chair. If Freud directed his gaze diagonally across the room into his office, his study chair was directly in his line of vision (see Figure 3). Perceptual lines and strategic object orientation defined Freud’s and the Egyptian’s architectural work. The perceptual axis lines generated by the positioning of Freud’s consulting room couch and chair produced the motif of the double. The doubles found in Freud’s office contributed to the sense of security, familiarity, and safety inherent to the active room.

## Couch as Active Container

**FIGURE 4:**
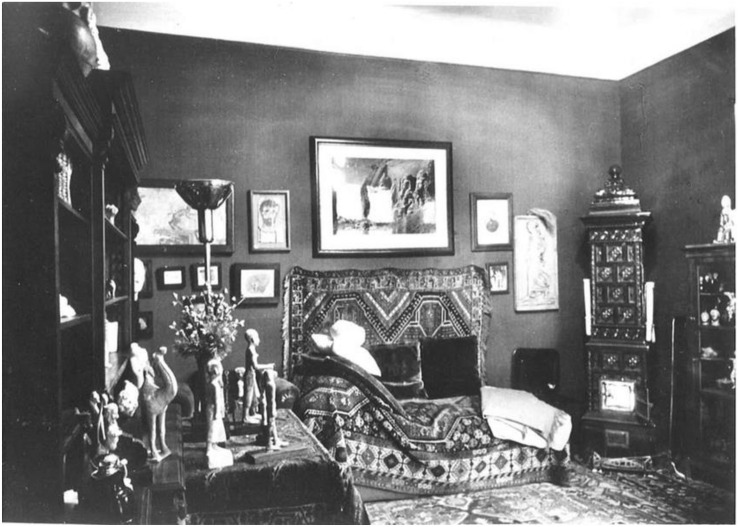
In Freud’s office, the patient’s initial disorientation was countered by the comfort provided by the haptic rugs, symmetric room composition, and couch. (Figure 4 by Edmund Engelman, courtesy of Thomas Engelman).

In Freud’s office, the patient’s initial disorientation was countered by the comfort provided by the haptic rugs, symmetric room composition, and couch (see Figure 4). Danze describes the patient’s experience on the couch in detail:

Once the analysand is invited to lie on the couch, in the reclining position, disorientation, reconfiguration and instability all naturally and fundamentally ensue. The shift from being grounded, upright, mobile and physically in control, from being in command of one’s physical location, to being passive, at rest … is one of the most subtle and powerful physical adjustments that initiates transformation (Danze, 2005, p. 113).

The haptic experience facilitated the patient’s acclimation to his new position on the couch. As previously alluded to, Freud’s couch symbolized the Egyptian sarcophagus. In 18th Dynasty tombs, the sarcophagus was traditionally positioned against the burial chamber wall. Freud’s couch was also positioned against the wall. Freud’s couch and the sarcophagus held and safeguarded the shrouded bodies.

Freud’s couch and the coffin denote boxes–active containers–that held and preserved the resting body as the inner being embarked on a psychic, spiritual journey into the afterlife. According to Freud, as the patient lay on the couch, “the ‘upward drive’ of the repressed stirred into activity” (Freud, 1937, p. 267). The analyst’s couch and its placement within Freud’s office contributed to the room’s status as an active agent within the psychoanalysis session. Reflecting on Freud’s psychoanalysis techniques, Danze concludes that the patient’s “new position and point of view [on the couch] transform the consulting room from a passive spectator to a full participant of the analytic work or from a neutral film to a receptive container of actions and movements” (Danze, 2005, p. 113). The furniture’s architecture contributed to the room’s initiation of the analysand’s and analyst’s psychic journey.

## Freud’s Chair

**FIGURE 5:**
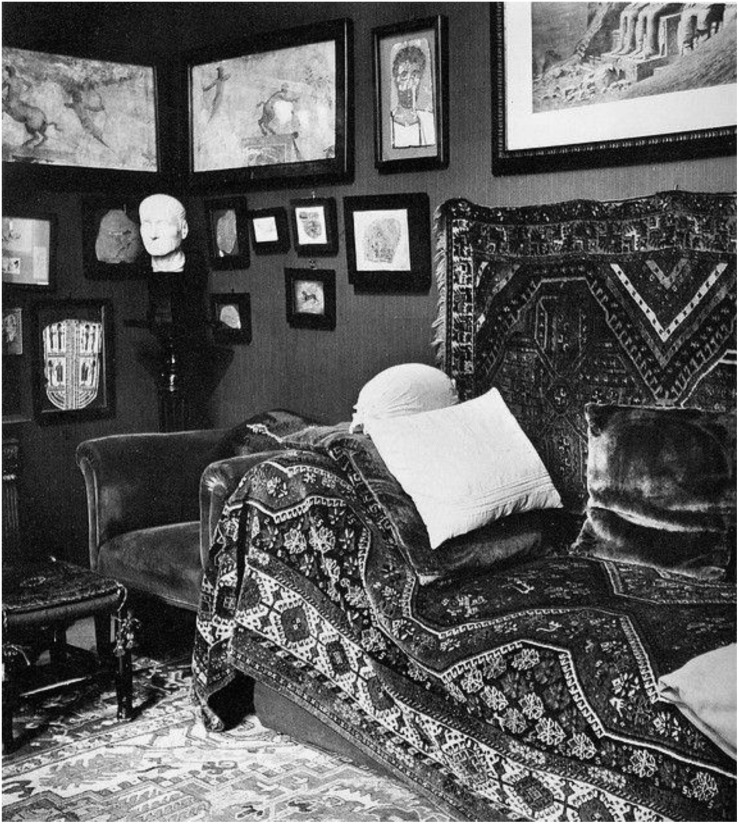
Freud assumed a passive position as he sat in his large, comfortable, reclining chair situated behind the couch. (Figure 5 by Edmund Engelman, courtesy of Thomas Engelman).

During psychoanalysis sessions, Freud also embarked on a psychic journey. Emulating the patient, he assumed a passive position as he sat in his large, comfortable, reclining chair situated behind the couch (see Figure 5). The spatial configuration embodies the active container’s primary facet: the patient “lies down and the analyst sits behind him” (Quinodoz, 1992, p. 630). Seated in the room’s corner, Freud was outside of the patient’s line of vision. Here, Freud leaned back and assumed a resting position. While the room remained active, the analyst played a passive role during psychoanalysis sessions. During sessions, Freud practiced a passive technique, which he termed “evenly-suspended attention.”

In “Recommendations to Physicians Practicing Psychoanalysis,” Freud describes the technique and his role within the contained office space. He recommends the analyst “should simply listen, and not bother about whether he is keeping anything in mind” (Freud, 1912, p. 77). Danze points out that “when the analysand is looking up, out, or over, and looking not at any particular focal point, there is made to exist a spatial openness, an implied spatial potential for infinity” (Danze, 2005, p. 112). Both chair and couch permitted the bodies to lean back comfortably and submit themselves to the forces of their unconscious.

## Active Room as Womb

The couch, coffin, and chair may also be interpreted as active containers. These vessels shelter and contain the body as it undergoes transformative processes. Each contained space was an “envelope and backdrop for what goes on in it, a sensitive vessel that holds all actions and movements, containing and holding the patient” (Danze, 2005, pp. 113–114). The office itself represented a maternal, comforting space. Winnicott’s description of the psychoanalyst’s need to create a maternal “holding environment” resonates with Freud’s clinical setting (Winnicott, 1965, p. 239). For Freud, the “dwelling-house was a substitute for the mother’s womb, the first lodging … in which he was safe and at ease” (Freud, 1930, p. 152). Herein lies a semiotic similarity between the languages of architecture and psychoanalysis. Freud’s office appears analogous to the womb. The office matured the patient until he was ready to move on from the maternal holding space.

Inside the womb and the tomb, the bodies were subject to forces beyond their control as they underwent formative transformations. Each holding vessel evokes the active container and active room frameworks. According to Danze’s “active room” theory, “the room itself has the power to initiate and sustain a shift in the analysand … The analytic space is then both a natural witness and powerful participant in the psychoanalytic relationship” (Danze, 2005, p. 110).

## Walls

In the active room, the walls create an enclosed space and provide a thinking interface for the patient. According to psychoanalyst Julie Leavitt, upon the interface “echoes of memories, the history, and the transference interact with the very physical dimension and make live and use again the visible features of the analytic room” (Schinaia, 2018, p. 181). The walls enclosing Freud’s office space created an interface for memories to play out on. The patient conjured up associations and connections as he gazed at Freud’s walls. Significantly, a large color print of Ramse’s temple at Abu Simbel with its colossal seated deities emerging out of a rock wall was located on the wall directly above the couch. Various memories full of hidden meanings would arise in the patient, who was positioned facing this wall. Freud’s patient Hilda Doolittle (H.D.) described the print in her *Tribute to Freud*:

In one of our talks in the old room at Berggasse, we had gone on one of our journeys. Sometimes the Professor knew actually my terrain, sometimes it was implicit in a statue or picture, like that old-fashioned steel engraving of the Temple at Karnak [the Ramse’s temple print] that hung above the couch. I had visited the particular temple, he had not (Doolittle, 1956, p. 9).

In a sense, the walls served as a memory substrate, reminiscent of Freud’s descriptions of the “mystic writing pad.” The psyche projected itself onto the physical environment.

In Egypt, “so much of the daily life [was] to be represented on the walls of the tomb chapel” (Smith and Simpson, 1999, p. 1). The walls preserved the mummy’s past life and memories, which would accompany him into the afterlife. To ensure that the past was preserved on the tomb walls, in ancient Egypt “enduring stone forms were chiefly reserved for the building of temples and tombs” (Smith and Simpson, 1999, p. 1). The strong stone walls “lead to the excellent preservation of so much which represents the peculiarly Egyptian emphasis upon tomb architecture” (Smith and Simpson, 1999, p. 1). The walls in Freud’s office and the Egyptian tomb functioned as a means of memory activation and preservation. The active room’s walls had the “capacity to evoke different kinds of associations” (Danze, 2005, p. 123). The psyche may be interpreted as having been preserved within the room’s physical structures, including the walls and terra cotta statues.

## Terra Cotta Statues

The Egyptians believed that after death the body was transfigured into the “ka,” an emanation of the spirit. The mummy’s ka had a separate, spiritual existence from the body and was described as the mummy’s “double or as a protective genius” (Smith and Simpson, 1999, p. 5). Resonating with Freud’s unconscious–conscious dualism, the Egyptians believed that human beings possessed two bodies–a spiritual one and a physical one. The terra cotta tomb statues served as the physical double of the ka. The terra cotta statues were traditionally placed near the sarcophagus. If the body was stolen, the statues stood in for the body.

André Bazin describes the significance of the Egyptian statues’ placement in detail: “the Egyptians placed terra cotta statuettes, as substitute mummies … It is this religious use, then, that lays bare the primordial function of statuary, namely, the preservation of life by a representation of life” (Bazin, 1960, p. 5). Bazin terms the immortality made possible by the mummy’s terra cotta double the “mummy complex.” Similarly, in “The Uncanny,” Freud claimed the ancient Egyptian “‘immortal’ soul was the first ‘double’ of the body” (Freud, 1919, p. 9). Freud asserted that “this invention of doubling as a preservation against extinction has its counterpart in … the Ancient Egyptians … art of making images of the dead in lasting materials” (Freud, 1919, p. 9). We may read this as a reference to the terra cotta statues created by the ancient Egyptians.

**FIGURE 6:**
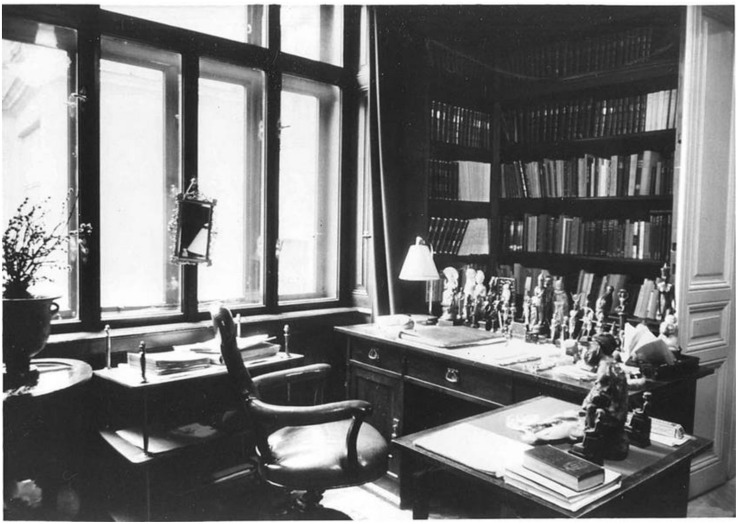
In Freud’s office study, Egyptian terracotta statues were lined up across his desk. A mirror was framed by Freud’s window. (Figure 6 by Edmund Engelman, courtesy of Thomas Engelman).

In Freud’s office study, Egyptian terracotta statues were lined up across his desk (see Figure 6). In his lectures, Freud describes his desk’s topographical arrangement as “a circle of bronze statuettes with small terra-cotta figures … set behind this inkstand” (Freud, 1901, p. 54). Freud emphasizes the statuette’s strategic placement by stressing their designated space “behind” the inkstand. A diagonal axis line drawn between the study and consulting room would appear to connect the statuettes standing atop Freud’s desk to the patient atop Freud’s consulting room couch. The statues appear to symbolize doubles of the patient. The statues’ positioning recalls that of the statues found near the sarcophagus and mummy in the ancient Egyptian tomb.

The statues’ positioning within the active room could be interpreted as permitting the patient’s doubling and rebirth. In other words, the statues served as the patient’s double as he was reborn during the psychoanalysis session. The statues aided Freud’s patients as they embarked upon their spiritual journey into the next enlightened life. These figurines played an active role within psychoanalysis sessions. In Charles Rice’s study entitled “Lost Objects: Sigmund Freud’s Psychoanalytical Interior,” the active role of Freud’s statues is also investigated (Rice, 2006, p. 46). Rice writes the antiquities may be seen as “elements of a patient’s psychological history” (Rice, 2006, p. 46). He also writes that the antiquities speak to the analyst, who must translate what is said into something meaningful. Rice’s claims further supplement the argument that Freud’s statues represent the patient’s double.

The active participation of the statues and their role as the patient’s double were also emphasized by Freud’s tendency to interact with his statues during psychoanalysis sessions. In her memoirs, Freud’s patient Hilda Doolittle writes, “we looked over the images in one of the other cases … The Professor brought out a wooden Osiris … blackened by time … there was another green-blue Osiris. The Professor said, ‘They are called the answerers, as their doubles or ka’s come when called”’ (Doolittle, 1956, p. 172).

Freud associates the double not only with the Egyptian tomb’s plastic arts but also with the mirror. Drawing upon Otto Rank, Freud examines “the connections the ‘double’ has with reflections in mirrors, with shadows, guardian spirits, with the belief in the soul and the fear of death” (Freud, 1919, p. 9). The intimate relationship between the double and the mirror may be traced back to the ancient Egyptian tomb.

## Mirrors

The mirrors in Freud’s office appear to provide a further parallel to the historically Egyptian motif of the double. Historically, “the Egyptians may have believed that the mirror helped preserve the ka, the double discovered in the mirror’s depths, and allowed it to make a transition to another life. Thus, mirrors are frequently depicted on the wall paintings directly before the face of the deceased” (Pendergrast, 2009, p. 5). In ancient Egypt, such mirrors were considered light reflectors that guided the way into the afterlife.

**FIGURE 7:**
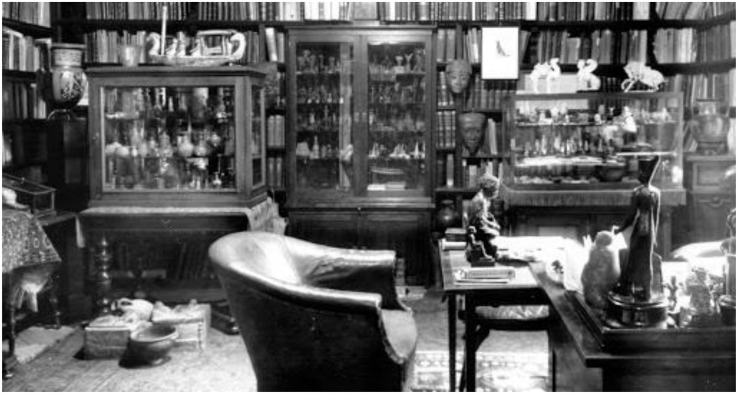
During initial consultation meetings, Freud’s patients sat directly across from Freud and the mirror. (Figure 7 by Edmund Engelman, courtesy of Thomas Engelman).

In Freud’s study, one mirror was positioned directly behind his desk. This mirror was framed by Freud’s window (see Figure 6). During initial consultation meetings, Freud’s patients sat directly across from this mirror (see Figure 7). The mirror framed the patient’s face and created his double while he sat in his designated position across from Freud.

Freud’s second mirror was placed directly across from his desk. When directing his gaze straight ahead, Freud encountered his own double (see Figure 3). This physical setup was perhaps intentional. In “Papers on Technique,” Freud claimed that “the doctor … should be opaque to his patients and, like a mirror, should show them nothing but what is shown to him” (Freud, 1914, p. 118). Freud often remained opaque and expressionless during consultation meetings, recalling the mummy’s death mask in ancient Egypt. The purpose of the Egyptian death mask painted onto the sarcophagus was “to provide a face for the dead in the afterlife” (Bryant and Peck, 2009, p. 322).

## The Active Room as a Tomb

Freud’s writings indicate his interest in the Egyptian afterlife. In his lectures, Freud asserts:

Every person acquainted with ancient rite knows how seriously, for example, the Egyptians considered the portrayal of a journey to the land of the dead. There still exist many copies of the “death book” which was given to the mummy for this journey as a sort of Baedeker. Since the burial places have been separated from the living quarters, the last journey of the dead person has become a reality (Freud, 1917, p. 126).

Here, Freud emphasizes the importance of isolating the tomb as a prerequisite for the Egyptian final journey to begin. Upon his death, Freud spent his final days in his tomb-like office, which he recreated in London at the end of his life.

In 1938, Freud fled Nazi-occupied Vienna. Freud reconstructed his tomb-like office in London with the help of his architect son, Ernst Freud. To “recreate the interior of Sigmund Freud’s Viennese study and consulting room was … [a] goal of the remodeling of 20 Maresfield Gardens” (Welter, 2011, p. 151). In order to maintain the previous office’s architectural composition, Ernst Freud “knocked together” two rooms by removing a wall (Welter, 2011, p. 151). Although Freud’s office no longer exhibited a central open doorway, the space remained composed of two conjoined rooms. Similar to Berggasse 19, the new office at 20 Maresfield Gardens remained separated from the Freud family’s living quarters. Fuss and Sanders note that Freud’s maid Paula Fichtl arranged the London office “to reproduce, as closely as possible, the office at Berggasse 19” (Fuss and Sanders, 2015, p. 15). For the most part, Freud’s office retained its previous spatial and architectural composition.

Freud only disbanded his clinical practice in London 2 months before his death. “During the final days, Freud requested that his bed be brought down to the study so that he could be near his books, desk, and beloved collection of antiquities” (Cohen, 2014, p. 3). In Freud’s tomb-like office, he assumed the role of the mummy. Freud quietly died on September 23, 1939. Freud was embalmed “in a tomb he spent over 40 years preparing” (Fuss and Sanders, 2015, p. 15). The preservation of Freud’s ashes in his London office amplifies this claim. Freud’s Egyptian tomb-like office immortalized his collection and legacy. Freud’s active room preserved his patient’s memories and his own. Drawing upon Diana Fuss and Joel Sanders, Charles Rice writes: “‘The location of the analytic scene within the four walls of a crypt … at Freud’s office … was an overdetermined space of loss and absence, grief and memory, elegy and mourning’ … This … was the spatialization of Freud’s own psychological interiority” (Rice, 2006, p. 43).

## Discussion and Wider Discourse

The notion that architecture represents a spatialization of the psychological interiority is further reflected in works that posit architecture’s status as an active, psychic entity. Therefore, the present study may also be seen as part of the wider discourse around the psychic potential of architecture. Contemporary architectural theorists, such as Juhani Pallasmaa and Lucy Huskinson, reference architecture’s active agency and psychic potential. These theorists oppose those who state that the therapeutic space (in the physical sense) should be neutral.

Those in support of the therapy room’s neutrality argue that personalized office design and architecture could “give indications of the analyst’s personality and interface with the analysand’s [patient’s] phantasmatic and associative freedom” (Schinaia, 2018, p. 194). Expanding upon this viewpoint, psychoanalyst Christopher Bollas writes that “few physical distractions” permit the self “to recline into [the mind’s] interiority” (Bollas, 2012, p. 53). Neutral therapy spaces are often characterized by “modern architecture,” “reduction, simplification, and deprivation (this idea is based on the German architect Ludwig Mies van der Rohe’s maxim *less is more*)” (Schinaia, 2018, p. 190). These spaces generally include “an armchair, a couch, sometimes a writing desk and closet” (Schinaia, 2018, p. 190). The tension between those who assert that the therapeutic space should be neutral and those who believe in the psychic agency of space may be considered a reaction to the “‘too full spaces’ of Freud’s and [the] first Freudian analysts’ rooms” (Schinaia, 2018, p. 190).

Today’s analysts seem to have renounced the “neutrality that was recommended until a few decades ago … in their exterior settings” (Bolognini, 2010, p. 8). Juhani Pallasmaa “opposes the notion of (supposed) neutrality of the environment and supports the need to ‘re-sensualise’ architecture” (Schinaia, 2018, p. 159). Pallasmaa asserts “profound architecture makes us experience ourselves as complete embodied and spiritual beings” (Pallasmaa, 2012, p. 13). Along a similar vein, Lucy Huskinson writes: “Buildings are active participants in the creation and development of personality” (Huskinson, 2018, p. 15). Citing Gaston Bachelard, whose book *The Poetics of Space* was formative in forging the relationship between psychoanalysis and architecture, Huskinson states: “‘Everything comes alive when contradictions accumulate’ … Through tensions of opposites the correspondence between psyche and place … is consolidated, with both aspects undergoing some sort of ‘expansion’ or enrichment as a result” (Huskinson, 2018, p. 135). In the office and tomb, the resting body could be viewed as undergoing a psychic expansion and enrichment. Therefore, we may situate the present analysis within the wider discourse around the active, psychic potential of architecture.

This study may also be productive to read in dialogue with the work of Diana Fuss and Joel Sanders. As previously stated, this study builds upon the article “Berggasse 19: Inside Freud’s Office” by Diana Fuss and Joel Sanders. Although Fuss and Sanders do not put the Egyptian office elements and architecture into the foreground and illuminate the office more as Freud’s own tomb, rather than a place where the patient undergoes a tomb-like transformation, their allusions to tombs strengthen the present argument. Whereas Fuss and Sanders explore “the role of both vision and hearing in three-dimensional space, examining how architecture organizes the physical and sensory interaction of bodies as they move through the interior of Freud’s study and consulting room,” the present study seeks to emphasize the active role the Egyptian architecture and elements played within Freud’s tomb-like office (Fuss and Sanders, 2015, p. 1). Both studies ultimately seek to shed light on the significant role Freud’s office architecture played in his psychoanalytic practice.

## Concluding Remarks

**FIGURE 8:**
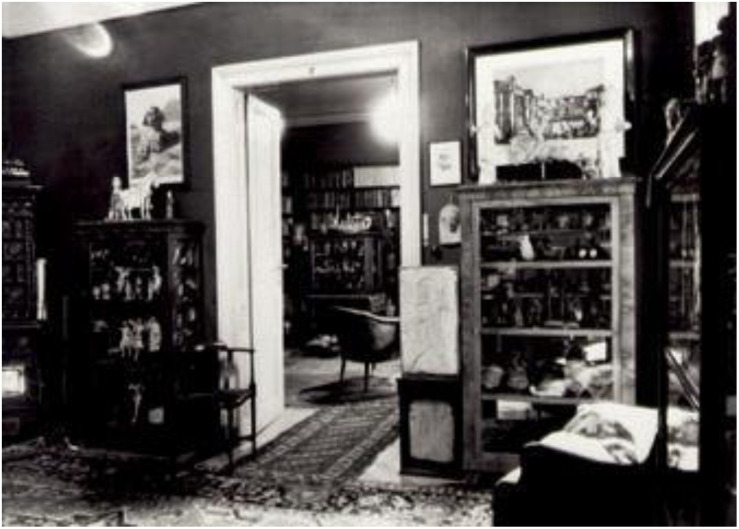
The office space at Berggasse 19 in Vienna actively influenced Freud’s practice, work, and legacy. (Figure 8 by Edmund Engelman, courtesy of Thomas Engelman).

The office space actively influenced Freud’s practice, work, and legacy (see Figure 8). In this study, I posit and provide support for the notion that the ancient Egyptian tomb elements and architecture in Freud’s office actively contributed to his psychoanalysis practice. Freud’s office and the ancient Egyptian tomb represent active rooms. These closed-off spaces significantly contributed to the contained individual’s psychic transformation. These active containers facilitated psychic transformations by liberating the mummy’s spirit and the patient’s unconscious. In our present historical moment, Freud’s office design continues to influence the architectural design of many psychoanalytic offices around the world. The psychoanalyst’s office remains an active vessel that facilitates transformation.

## Author Contributions

The author confirms being the sole contributor of this work and has approved it for publication.

## Conflict of Interest

The author declares that the research was conducted in the absence of any commercial or financial relationships that could be construed as a potential conflict of interest.
